# Instructions for authors (www.scielo.br/spmj)

**DOI:** 10.1590/S1516-31802008000400014

**Published:** 2008-07-03

## Aim and editorial policy

The São Paulo Medical Journal - Evidence for Health Care, founded in 1932, is one of the oldest medical publications in Brazil. Its articles are indexed in Medline, Lilacs and SciELO. Published bimonthly by the Associação Paulista de Medicina, the journal accepts articles in the field of clinical health science (internal medicine, gynecology and obstetrics, mental health, surgery, pediatrics, and public health). Articles will be accepted in the form of original articles (experimental studies), literature reviews or updating papers, case reports, short communications and letters to the Editor. Papers with a commercial objective will not be accepted.

## Procedures of the journal

After receipt of the article by the Publications Unit, the authors will be provided with a protocol number. This number serves to maintain good understanding between the authors and the Publications Unit. Following this, the article will be read by the Editor, who will verify whether it is consonant with the journal's policy and interests, i.e. whether the field of the research or review is within the areas of health or public health.

Next, the Publications Unit will verify whether the text complies with the journal's Instructions for Authors. If the text is incomplete or if it is not organized as required, the authors will be asked to resubmit their text after resolving such problems. When its form is acceptable, the Publications Unit will submit the manuscript to open peer review (unless reviewers declare that they prefer closed peer review).

Open peer review means that reviewers sign their judgment and that they know the names of the authors. The reviewers are professionals or researchers working in the same field as dealt with by the manuscript. The main objectives of the review are to detect problems in the methodological design and see whether the conclusions are focused on the results presented.

Authors will then receive the reviewers’ judgment and will be asked to resolve all the problems pointed out. Once the Publications Unit receives the manuscript again, the text will be sent to the scientific editor and the proofreader, who will point out problems with phrase construction, spelling, bibliographical references and others. Authors should then provide all further information required.

When the text is considered acceptable for publication, and only then, it will enter the queue for publication. The Publications Unit will provide a proof, including any tables and figures, for the authors to approve. No article is published without this last procedure.

## Instructions for authors

### The manuscript and types of articles

The manuscript must be sent in English. Nonetheless, it must also include a summary and five key words both in Portuguese (or Spanish) and in English. Texts may be sent in digital form (3 1/2” disk/CD), in “.doc” or “.rtf” extensions (no other will be accepted) with one printed copy, to the Publications Unit (at Av. Brigadeiro Luís Antônio, 278 - 7o andar - CEP 01318-901, São Paulo/SP), or via the internet, to revistas@apm.org.br.

Papers submitted must be original and be accompanied by a declaration, signed by all the authors, that the text has not and will not be published in any other journal. Research articles involving human beings must be submitted together with a copy of the authorization from the Ethical Committee of the institution in which the work was performed.

Papers submitted must comply with the editorial standards established in the Vancouver Convention (Uniform Requirements for Manuscripts Submitted to Biomedical Journals)^1^ and the rules for reports on clinical trials,^2^ and systematic reviews.^3^

The Sao Paulo Medical Journal supports the clinical trial registration policies of the World Health Organization (WHO) and the International Committee of Medical Journal Editors (ICMJE) and recognizes the importance of these initiatives regarding the registration and internationally dissemination of information on clinical studies, with open access. Thus, from 2008 onwards, clinical research papers will only be accepted for publication if they have received an identification number from one of the Clinical Trial Registers that have been validated in accordance with the criteria established by WHO and ICMJE. The adresses for these registers are available from the ICMJE website (http://www.icmje.org). The identification number should be declared at the end of the abstract.

The paper (original articles, short communications and case reports) must be structured so as to contain these parts: introduction, methods, results, discussion and conclusion. Literature reviews may be freely structured, but the text must contain a final part for a conclusion or final considerations.

Abbreviations may not be used, even those in common use. Drugs must be referred to by their generic names, avoiding unnecessary mention of commercial or brand terms. Any product cited in the Methods section, such as diagnostic and testing equipment, reagents, instruments, utensils, prostheses, orthoses and intraoperative devices must be described together with the manufacturer's name and place of manufacture. Medications administered must be described using their generic names followed by the dosage used and posological data.

Grants and any other financial support for the work must be mentioned separately, on the first page. Acknowledgments, if necessary, must be placed after the references.

### Original articles (experimental studies)

The text must not exceed 5,000 words (excluding tables, figures and references) and must include a structured abstract with a maximum of 250 words.^4^ The structure of the text should whenever possible follow the format laid out below:

1. *Introduction:* specify the reasons for carrying out the study, describing the present state of knowledge of the theme. Do not include here any results or conclusions of the study. Use the last paragraph to specify the principal question of the study, and the principal hypothesis tested, if there is one.
2. *Objective:* described briefly what the main objective of the study was.
3. *Methods*
  *3.1) Type of study:* describe the design of the study specifying, if appropriate, the use of randomization, blind studies, diagnostic test standards and the time direction (retrospective or prospective). For example: randomized clinical trial, double-blind, controlled placebo, or accuracy study.
  *3.2) Setting:* indicate where the study was carried out, including the healthcare ranking (for example: primary or tertiary; private or public institution).
  *3.3) Sample (participants or patients):* describe the selection procedures, inclusion criteria and the number of patients at the beginning and end of the study.
  *3.4) Procedures (intervention, diagnostic test or exposition, if necessary):* describe the principal characteristics of any intervention, including the method and duration of its administration.
  *3.5) Main measurements:* describe the method of measuring the primary result, in the way it was planned before data collection. If the hypothesis reported was formulated during or after data collection, this fact needs to be specified.
  *3.6)Statistical methods:* describe the sample size calculation method, the planned statistical analysis, statistical tests used and significance levels, and some post hoc analysis.
4. *Results:* describe the principal results. If possible, these should be accompanied by their 95% confidence interval and the exact level of statistical significance. For comparative studies, the confidence interval must be stated for the differences between the groups.
5. *Discussion:* emphasize the new and important factors encountered in the study, which will form part of the conclusion. Do not repeat data presented in the introduction or results in detail. Mention any limitations of your findings that should be noted and possible implications for future research. Relate any observations from other relevant studies.
6. *Conclusions:* specify only the conclusions that can be sustained by the results, together with its clinical significance (avoiding excessive generalization), or whether additional studies would be necessary before the information could be put into practice. The same emphasis should be placed on studies with positive and negative results.

### Short communications or case series and case reports

Short communications and case reports must be limited to 1,000 words and five references, including an abstract with a description of the case and a pertinent discussion, and key words. The text must be divided in parts as described for original articles, and references in the Vancouver Style.^1^

### Review or updating articles

Review and updating articles have free format, provided they present references in the Vancouver Style.^1^. Systematic review articles or meta-analyses must comply with the rules for reports on systematic reviews.^3^ The text must not exceed 5,000 words (excluding tables, figures and references).

### Research letters

All research letters published are peer reviewed, with separate statistical review where appropriate. *The São Paulo Medical Journal - Evidence for Health Care* wishes to be flexible but ideally a research letter should have no more than 900 words, and a maximum of five references and two tables or figures. An unstructured summary of not more than 100 words is required. The signatures and description of the contributions of all authors are required. A proof will be provided, usually by fax, and the authors must respond immediately because the journal may wish to publish quickly. Authors will be told of non-acceptance.

### Letter to the editor

In this category the text must not exceed 500 words and five references.

## Format

### First page

The first page must contain: 1) the title of the paper in English and Portuguese (or Spanish), which must be short but informative; 2) the type of paper (original article, review or updating article, short communication, letter to the editor); 3) the name of each author (do not abbreviate), his/her highest academic title attained and the institution where he/she works; 4) the place where the work was developed; 5) the meeting, date, and place where the paper was presented, if applicable; 6) the complete address, e-mail and telephone number of the author to be contacted by the Publication Unit regarding the manuscript; and the addresses and telephone numbers of the main author for publication (which may or may not be the same); 7) sources of support in the forms of finance, equipment or drugs, and the grant numbers; 8) description of any conflicts of interest held by the authors.

### Second page: abstract and key words

The second page must include an abstract^4^ structured in parts in accordance with the classification of the article. For original articles, there are five items: 1) context and objective; 2) design and setting (where the study was performed); 3) methods (described in detail); 4) results; and 5) conclusions.

This page should also contain five key words. These English terms must be chosen from the Medical Subject Headings (MeSH) list of Index Medicus, which is available on the internet.^5^

### References

The references (in the “Vancouver style”, as stated by the International Committee of Medical Journal Editors, 1997) should be laid out on the final pages of the article and numbered in the order of citation. References cited in legends of tables and figures must maintain sequence with references cited in the text. All the authors must be listed if there are less than six; if there are six or more, the first three should be mentioned and followed by “et al.”. Some examples of the most common types of references:

### Article in journal

– Lahita R, Kluger J, Drayer DE, Koffler D, Reidenber MM. Antibodies to nuclear antigens in patients treated with procainamide or acetylprocainamide. N Engl J Med 1979;301:1382-5.

### Chapter of book

– Reppert SM. Circadian rhythms: basic aspects and pediatric implications. In: Styne DM, Brook CGD, editors. Current concepts in pediatric endocrinology. New York: Elsevier; 1987.p.91-125.

### Text on the internet

– Morse SS. Factors in the emergence of infectious diseases. Available from http://www.cdc.gov/ncidod/EID/eid.htm. Accessed in 1996 (Jun 5).

### Last page

The last page must contain an abstract written in Portuguese or Spanish, followed by at least five key words (“palavras chave”) chosen from among the Subject Descriptors created by Bireme, which are available on the internet.^6^

### Figures and tables

Images must have good resolution (minimum of 300 DPI) and be recorded in “.jpg” or “.png” format. Do not attach images inside Microsoft PowerPoint documents. If photographs are attached to a Microsoft Word file, send the images separately as well. Graphs must be prepared in Microsoft Excel (do not send them in image formats) and must be accompanied by the tables of data from which they have been generated. The number of illustrations must not exceed half the total number of pages minus one.

All figures and tables must contain legends or titles that precisely describe their contents and the context or sample from which the information was obtained (i.e. what the results presented are, what the kind of sample or setting was). The legend or title sentence should be short but comprehensible without depending on reading the article.
